# Divergent Responses of Community Reproductive and Vegetative Phenology to Warming and Cooling: Asymmetry Versus Symmetry

**DOI:** 10.3389/fpls.2019.01310

**Published:** 2019-10-17

**Authors:** Fandong Meng, Lirong Zhang, Haishan Niu, Ji Suonan, Zhenhua Zhang, Qi Wang, Bowen Li, Wangwang Lv, Shiping Wang, Jichuang Duan, Peipei Liu, Wangmu Renzeng, Lili Jiang, Caiyun Luo, Tsechoe Dorji, Zhezhen Wang, Mingyuan Du

**Affiliations:** ^1^Key Laboratory of Alpine Ecology and Biodiversity, Institute of Tibetan Plateau Research, Chinese Academy of Sciences, Beijing, China; ^2^Graduate University of Chinese Academy of Sciences, Beijing, China; ^3^Key Laboratory of Adaptation and Evolution of Plateau Biota, Northwest Institute of Plateau Biology, Chinese Academy of Sciences, Xining, China; ^4^CAS Center for Excellence in Tibetan Plateau Earth Science, Chinese Academy of Sciences, Beijing, China; ^5^Binhai Research Institute in Tianjin, Tianjin, China; ^6^University of Chicago Medicine and Biological Sciences Division, Chicago, IL, United States; ^7^Institute for Agro-Environmental Sciences, National Agriculture and Food Research Organization, Tsukuba, Japan

**Keywords:** community phenological sequences, reciprocal transplant experiment, plant–climate interactions, temperature sensitivities, Tibetan Plateau

## Abstract

Few studies have focused on the response of plant community phenology to temperature change using manipulative experiments. A lack of understanding of whether responses of community reproductive and vegetative phenological sequences to warming and cooling are asymmetrical or symmetrical limits our capacity to predict responses under warming and cooling. A reciprocal transplant experiment was conducted for 3 years to evaluate response patterns of the temperature sensitivities of community phenological sequences to warming (transferred downward) and cooling (transferred upward) along four elevations on the Tibetan Plateau. We found that the temperature sensitivities of flowering stages had asymmetric responses to warming and cooling, whereas symmetric responses to warming and cooling were observed for the vegetative phenological sequences. Our findings showed that coverage changes of flowering functional groups (FFGs; i.e., early-spring FFG, mid-summer FFG, and late-autumn FFG) and their compensation effects combined with required accumulated soil temperatureto codetermined the asymmetric and symmetric responses of community phenological sequences to warming and cooling. These results suggest that coverage change in FFGs on warming and cooling processes can be a primary driver of community phenological variation and may lead to inaccurate phenlogical estimation at large scale, such as based on remote sensing.

## Introduction

Climate warming causes a series of ecosystem responses (Walther et al., 2002), including changes in plant phenology (Li et al., 2016; Körner, 2016). Phenological changes would have an important effect on the carbon cycle and ecosystem productivity (Wolkovich and Cleland, 2014). Long-term *in situ* or remote sensing observations and manipulative warming experiments are the main methods used in phenological studies at present (Arft et al., 1999; Walker et al., 2006; Cleland et al., 2007; Morisette et al., 2009; Pieper et al., 2011; Li et al., 2016). Some studies have found that phenological temperature sensitivities are mismatched at community and species levels (Steltzer and Post, 2009; Wolkovich et al., 2014; Meng et al., 2016; Meng et al., 2017). This mismatch may be caused by divergent responses of different species to warming (Steltzer and Post, 2009; Wolkovich et al., 2014). However, these studies only consider that warming is the principal factor, ignoring daily or annual frequent temperature fluctuations (Menzel et al., 2011; Kosaka and Xie, 2013). Ignoring cooling effects may lead to a biased prediction of phenology under the background of global warming. Several studies have found that warming and cooling exert different influences on plant phenology (Menzel et al., 2011; Wang et al., 2014a; Meng et al., 2016; Meng et al., 2017). For example, spring temperature variance reduces the temperature sensitivity of early phenophases (Wang et al., 2014c). This may be caused by a higher temperature sensitivity of phenology to cooling (8.4°C) than to warming (-2.1°C), whereas the opposite pattern has also been found for some species (Wang et al., 2014a). Moreover, some studies find that global annual mean temperature has frequently fluctuated (IPCC, 2007). For example, although the Tibetan Plateau is warming across the years, the annual average surface temperature at the Haibei Alpine Meadow Ecosystem Research Station (HBAMERS) at the Tibetan Plateau is 22 out of 44 years (from 1957 to 2000) lower than average, suggesting that warming and cooling spells have frequently occurred in this region (Li et al., 2004). However, the prevailing focus has been on the effect of warming on phenology, and few studies have distinguished differences in phenological responses to warming and cooling (Wang et al., 2014a; Meng et al., 2016; Li et al., 2016; Signarbieux et al., 2017). In particular, one study found a significant difference in the response of flowering to warming and cooling, which indicates an asymmetrical response, whereas a symmetrical response would be indicated by an equal magnitude of the response of phenology to warming and cooling on phenology (Wang et al., 2014a). However, there is little evidence to date on asymmetrical or symmetrical responses to warming and cooling for community phenological sequences.

Many studies have shown that phenological temperature sensitivity is species-specific, even under similar environmental conditions (Diez et al., 2012; Iler et al., 2013; Wang et al., 2014a, Wang et al., 2014b). Hence, phenological changes at the species level are difficult to match with changes at the community level due to divergent responses by different species (Steltzer and Post, 2009). However, few studies have focused on the compensatory effects of different species to community phenological sequences based on field observations. For example, individual species’ responses may be mutually offsetting if data on advanced and delayed phenophases of individual species are pooled (Meng et al., 2016; Meng et al., 2017). Especially, climate change is the main driver of biodiversity changes (Gill et al., 1998; Sala et al., 2000; Augspurger et al., 2005; Chuine, 2010; Wolf et al., 2017). Changes in community composition induced by temperature change would affect the responses of community phenological sequences to temperature change (Cleland et al., 2006; Cleland et al., 2007). In particular, community composition may have contrasting reactions to warming and cooling (Meng et al., 2017; Meng et al., 2018). Therefore, we hypothesized that changes in the coverage of flowering functional groups [FFGs; i.e., early-spring FFG (ESF), mid-summer FFG (MSF), and late-autumn FFG (LAF)] could be associated with the response patterns (i.e., asymmetry vs. symmetry) of community phenological sequences to warming and cooling.

In this study, we used a reciprocal transplant experiment to accomplish warming and cooling at four elevation gradients (i.e., 3,200, 3,400, 3,600, and 3,800 m a.s.l.) (Wang et al., 2014a). Our previous reports focused on changes in FFG coverage affecting community phenological sequences to warming and cooling (Meng et al., 2017). Here, we explore their response patterns to warming and cooling to test whether changes in temperature and FFG coverage codetermine asymmetric or symmetric responses of community phenological sequences to warming and cooling. Our objectives are to answer the following questions: (1) Are the responses of temperature sensitivities of FFG coverage and community phenological sequences to warming and cooling asymmetrical or symmetrical? (2) What are the main factors affecting their response patterns in the alpine region?

## Materials and Methods

### Design of the Reciprocal Transplant Experiment

**Figure 1:**
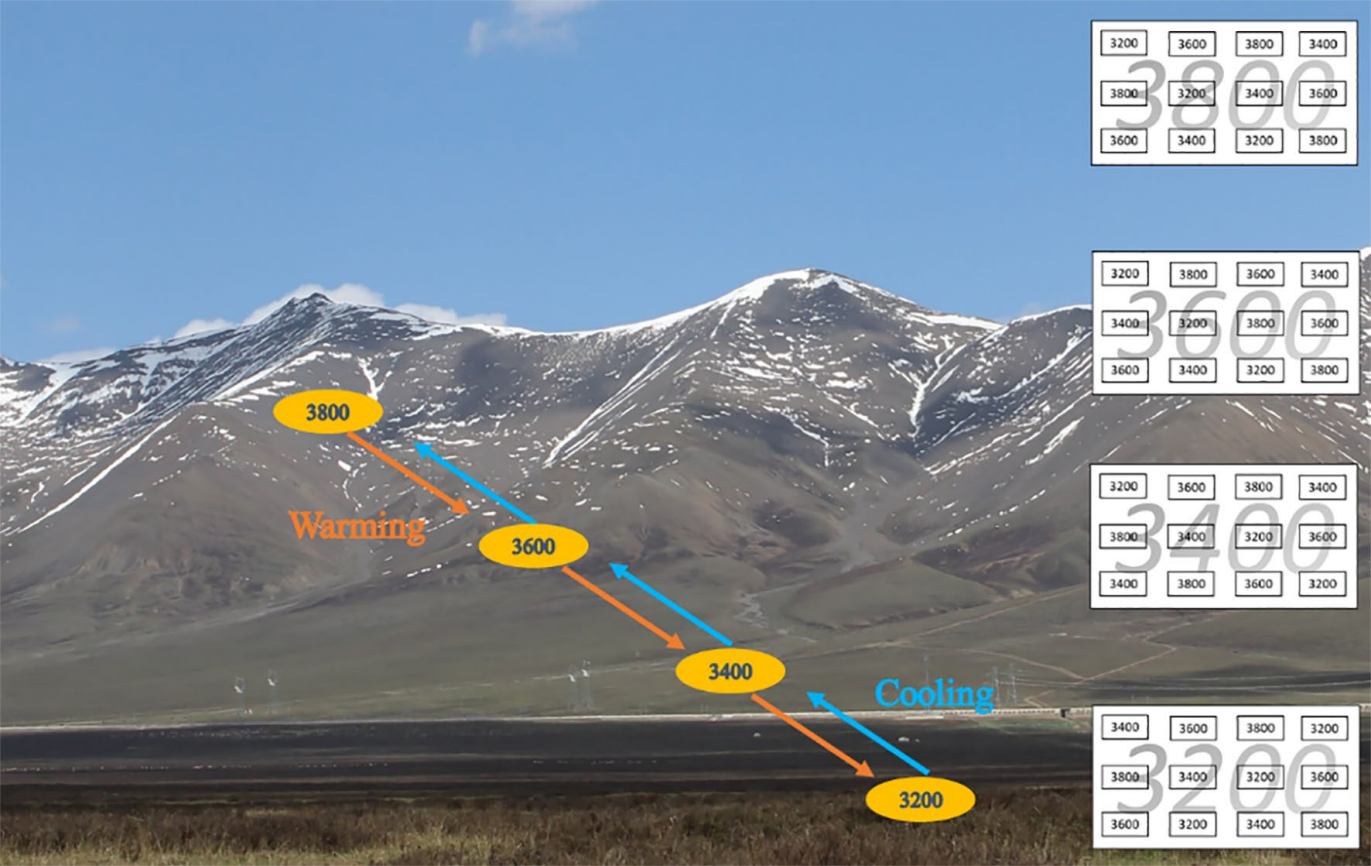
Reciprocal transplant experiment diagram and landscape. Each elevation has 12 plots (three plots per elevation). Small rectangles with black numbers indicate plots transferred from their elevations, and large rectangles with gray numbers indicate the elevation gradient. Red arrows indicate downward transfer (i.e., warming), and blue arrows indicate upward transfer (i.e., cooling).

The study site is located at HBAMERS (37°37′ N, 101°12′ E). Our experiment was carried out along four elevation gradients (i.e., 3,200, 3,400, 3,600, and 3,800 m a.s.l.), digging out 12 plots from each elevation (i.e., 48 plots in total) before soil thawing in May 2007. Each plot was 1 m × 1 m × 0.4 m (the depths at 3,600 and 3,800 m were 0.3 m due to their shallower soil layer; **Figure 1**). Then nine randomly selected plots at each elevation were reciprocally averagely transplanted to the other three elevations (i.e., three plots per elevation; **Figure 1**). For example, 9 of 12 plots from 3,200 m were randomly transplanted to 3,400, 3,600 and 3,800 m (i.e., three plots each elevation), leaving three plots in the original elevation (**Figure 1**); the other three elevations also had a similar distribution. Therefore, 12 plots of 3,200 m had three plots from 3,200 m, three plots from 3,400 m, three plots from 3,600 m, and three plots from 3,800 m (**Figure 1**). The spacing distance between each plot was 1 m. Grazing was excluded by fencing the plots at each elevation.

### Community Phenological Sequences, Coverage, and Meteorological Factor Monitoring

To observe community phenological sequences and coverage, a quadrant of 1 m × 1 m was used with 100 cross points (10 cm × 10 cm) for each plot. Phenological variation and coverage of each species were recorded under each cross point. The observation intervals of community phenological sequences were 3–5 days during each full growing season from 2008 to 2010. The observation of coverage was conducted once in late August or early September in each of the 3 years. Seven phenological sequences were monitored. The timings of community phenological sequences were the date on which the corresponding phenophase occurred for 15% of individuals, irrespective of plant species (Meng et al., 2017). The phenophases observed were onset of leaf out (OLO), first flower bud (FB), first flowering (FF), first fruiting set (FFS), postfruiting vegetation (OPFV), and first leaf coloring (FLC). The end of complete leaf coloring (CLC) was the date on which CLC occurred for 95% of individuals (Meng et al., 2016; Meng et al., 2017). All observed species were divided into three FFGs based on their flowering times (i.e., ESF, MSF, LAF; as detailed in Meng et al., 2017). Here, FFGs are defined as collections of organisms based on similar flowering behavioral or environmental responses. Plant species were classified into three FFGs (i.e., ESF, MSF, and LAF) based on their life history (Meng et al., 2016). Coverages of different FFGs were calculated for each plot in mid-August of each year based on the frequency of individuals of each FFG, irrespective of plant species, at the cross points of the 100 grid cells (Meng et al., 2016; Meng et al., 2017). Mean coverages of ESF and MSF across the natural elevation gradients (i.e., no translocation) were about 30% and 70%, respectively (**Table S1**, Meng et al., 2016). Meanwhile, coverages of dominant species were less than 50%, and there were more than 40 species in the natural community (**Table S1**, Meng et al., 2016).

Soil temperatures at 5-cm depth and moisture at 20-cm depth were monitored at an interval of 1 min by HOBO (Onset Computer Corporation, Cape Cod, Massachusetts, USA) weather stations at each site (Wang et al., 2014a). We compared Pearson’s correlation coefficients of community phenological sequences between air and soil temperature and found that soil temperature was a better predictor than air temperature (**Table S2**), as had been found in our previous study at species level (Wang et al., 2014a). Therefore, we chose soil temperature as our predictor variable.

### Data Analysis

We used a slope method to calculate the sensitivities of phenology and coverage (i.e., *a* coefficient based on y = a*x +b), where *y* and *x* are the differences between receptor and donor sites in community phenological sequences and soil temperature changes, respectively. Transfer downward and upward signified warming and cooling, respectively. Positive and negative values of the temperature sensitivities of community phenological sequences (CPS) represent delay and advance in days per 1°C change, respectively.

A general linear model was used to test the effects of treatments and their interactions on the coverage of different FFGs and the differences in phenological events in SPSS version 23. Analysis of covariance (ANCOVA) [in the form of lm(CPD∼Ts*grp)] was used to test the slope heterogeneity between warming and cooling. Here, CPD signifies the differences in date of CPS between transferred and control sites; Ts represents soil temperature differences between transferred and control sites; and grp represents the categorical variables warming and cooling. All functions and packages used were from R 3.3.3 (R Core Team, 2017). To assess the effects of coverage change on CPS, partial correlations between changes in CPS and coverage changes were calculated, setting temperature changes as the control variable. The required accumulated soil temperature (RCST) of CPS was defined as the sum of daily soil mean temperature above 0°C from 1 November of the previous year to a certain phenophase (Wang et al., 2014a). Chilling requirement (CR) was defined as the sum of daily soil mean temperature below 0°C from 1 November of the previous year to a certain phenophase.

## Results

### Asymmetric and Symmetric Responses of the Temperature Sensitivities of Different FFGs to Warming and Cooling

**Figure 2:**
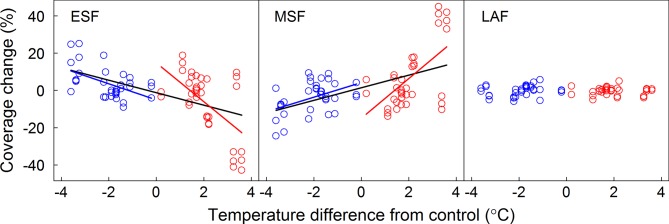
Relationships between coverage changes of FFGs and the annual temperature difference between receptor site and donor site. Linear regression equations for warming, cooling, and pooled warming and cooling data are indicated by red, blue, and black lines, respectively. ESF, early-spring flowering functional group; MSF, mid-summer flowering functional group; LAF, late-autumn flowering functional group.

Coverages of ESF and MSF were significantly affected by year, donor sites, receptor sites, and most of their interactions (**Table 1**). However, coverage of the LAF was only affected by donor site and interaction between year and donor site (**Table 1**). The temperature sensitivities of FFG coverage for ESF and MSF were -10.42 and 10.66% °C^-1^ under warming and 4.35 and -3.93% °C^-1^ under cooling, respectively (**Table S3** and **Figure 2**). When warming and cooling data were pooled, they were -3.35 for ESF and 3.39% °C^-1^ for MSF (**Table S3** and **Figure 2**). Furthermore, the regression models of LAF were nonsignificant under warming and cooling and with pooled warming and cooling data (**Table S3** and **Figure 2**). Based on the results of the ANCOVA test, slope values had significant differences between warming and cooling for ESF and MSF, but there were nonsignificant differences for LAF (**Table 2**). Thus, our results indicate that the temperature sensitivities of coverage of ESF and MSF had asymmetric responses to warming and cooling (**Table 2**).

**Table 1:** Analysis of variance of coverage of different flowering functional groups based on general linear models over 3 years.

Source	ESF			MSF			LAF		
	SS	df	P	SS	df	P	SS	df	P
Year (Y)	1,121.318	2	<0.001	988.157	2	<0.001	15.551	2	0.128
Donor (D)	3,230.945	3	<0.001	2,600.765	3	<0.001	41.657	3	0.015
Receptor (R)	1,089.35	3	<0.001	1,103.025	3	<0.001	16.368	3	0.225
Y * D	1,135.346	4	<0.001	883.559	4	0.002	76.049	4	0.001
Y * R	1,132.716	4	<0.001	1,215.588	4	<0.001	22.261	4	0.207
D * R	745.921	5	0.004	714.522	5	0.014	1.368	5	0.996
Y * D * R	213.169	2	0.066	194.682	2	0.123	0.855	2	0.889
Error	1,780.778	48		2,137.235	48		174.143	48	

ESF, early-spring flowering functional group; MSF, mid-summer flowering functional group; LAF, late-autumn flowering functional group.

**Table 2:** Slope heterogeneity of temperature sensitivities of different flowering functional groups and community level between warming and cooling.

	OLO	FB	FF	FFS	OPFV	FLC	CLC	ESF	MSF	LAF
PS or C	0.454	**0.014**	**0.009**	0.349	0.831	0.428	0.534	**0.026**	**0.016**	0.291

PS and C signify phenophases and coverage changes, respectively. P value in the ANCOVA method is the interaction effect in the test of the linear model. OLO, onset of leaf-out; FB, first bud/boot-set; FF, first flowering; FFS, first fruit set for forbs or seeding-set for graminoids; OPFV, onset of postfruiting vegetation; FLC, first leaf coloring; CLC, the date of complete leaf coloring; ESF, early-spring flowering functional group; MSF, mid-summer flowering functional group; LAF, late-autumn flowering functional group. Bolded text means 0.05 level.

### Asymmetric and Symmetric Responses of Temperature Sensitivities of CPS to Warming and Cooling

**Figure 3:**
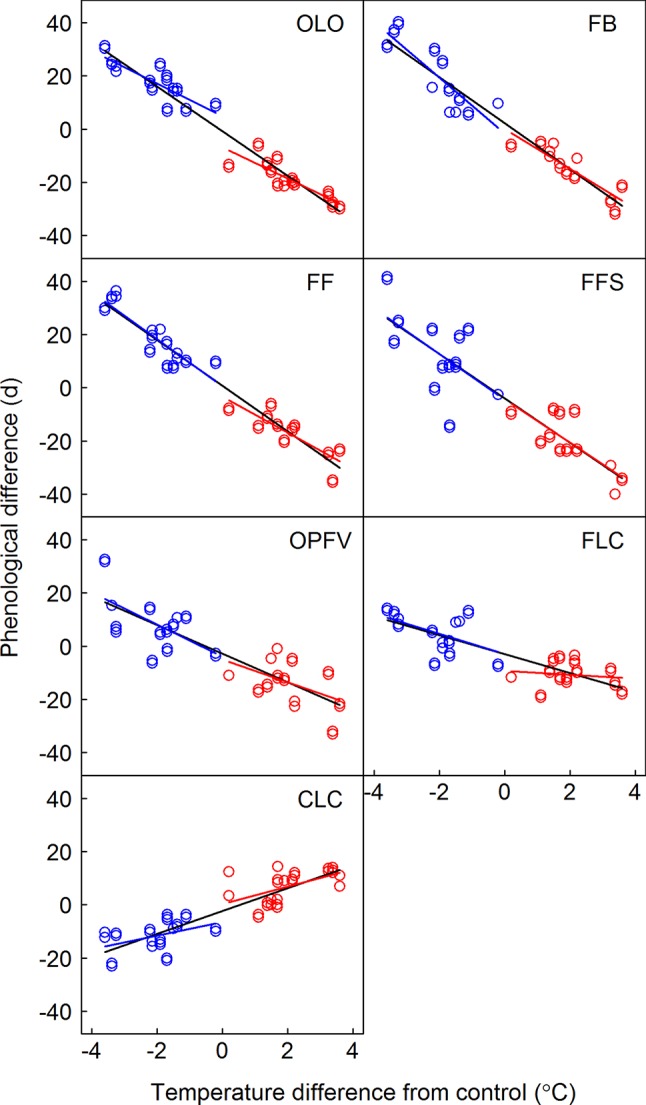
Relationship between the differences in timing of phenological sequences and temperature differences between receptor site and donor site. Linear regression equations for warming, cooling, and pooled warming and cooling data are indicated by red, blue, and black lines, respectively. OLO, onset of leaf-out; FB, first bud/boot-set; FF, first flowering; FFS, first fruit set for forbs or seeding set for graminoids; OPFV, onset of postfruiting vegetation; FLC, first leaf coloring; CLC, the date of complete leaf coloring.

Onset of the CPS was significantly affected by year (from 2008 to 2010), donor sites, receptor sites, phenophases, and their interactions (p < 0.001, **Table 3**). The temperature sensitivities were -5.8, -7.6, -6.7, -8.3, -4.1, -1.3, and 3.1°C under warming and 5.9, 10.3, 9.1, 7.8, 5.7, 3.5, and -3.0°C under cooling for OLO, FB, FF, FFS, OPFV, FLC, and CLC, respectively (**Table S3** and **Figure 3**). When warming and cooling data were pooled, the temperature sensitivities were -8.5, -8.5, -8.5, -8.6, -5.6, -3.7, and 4.3°C for OLO, FB, FF, FFS, OPFV, FLC, and CLC, respectively (**Table S3** and **Figure 3**).

**Table 3:** Analysis of variance of timing differences in community phenological sequences based on general linear models over 3 years.

Source	SS	df	Sig.
Year (Y)	963.954	2	<0.001
Donor (D)	29,563.690	3	<0.001
Receptor (R)	23,278.409	3	<0.001
Phenophase (P)	1,844.144	6	<0.001
Y * D	1,351.883	5	<0.001
Y * R	572.058	5	<0.001
Y * P	892.939	12	<0.001
D * R	416.331	5	<0.001
D * P	21,423.539	18	<0.001
R * P	14,686.850	18	<0.001
Y * D * R	583.069	6	<0.001
Y * D * P	1,789.599	30	<0.001
Y * R * P	2,448.979	30	<0.001
D * R * P	1,277.900	30	<0.001
Y * D * R * P	1,080.838	36	<0.001
Error	262.000	420	

Slope heterogeneities showed nonsignificant differences between warming and cooling for OLO, FFS, OPFV, and CLC, but there were significant differences for FB and FF based on the ANCOVA test (**Table 2**). Thus, our results indicate that all temperature sensitivities of vegetative phenophases and FFS had symmetric responses to warming and cooling, whereas the temperature sensitivities of flowering phenophases (e.g., FB and FF, **Table 2**) showed asymmetric responses to warming and cooling.

### Effects of Biotic and Abiotic Factors on Symmetric and Asymmetric Responses of the Temperature Sensitivities of CPS

**Figure 4:**
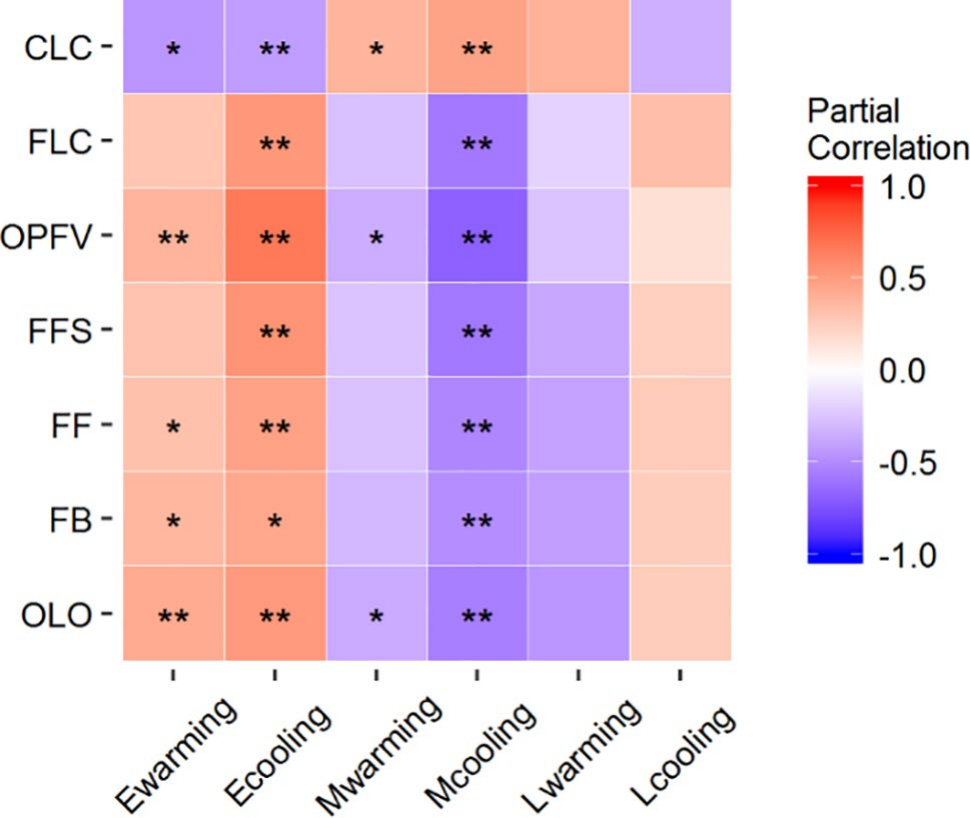
Partial correlations between coverage changes of FFGs and differences in CPS. *P < 0.05 and **P < 0.01. OLO, onset of leaf-out; FB, first bud/boot set; FF, first flowering; FFS, first fruit set for forbs or seeding set for graminoids; OPFV, onset of postfruiting vegetation; FLC, first leaf coloring; and CLC, the date of complete leaf coloring. Ewarming and Ecooling, Mwarming and Mcooling, and Lwarming and Lcooling signify coverage changes of ESF, MSF, and LAF under warming and cooling, respectively.

Nearly all coverage changes of ESF under warming and cooling and MSF under warming had significantly positive or negative correlations with CPS (**Table S4** and **Figure 4**). Only OLO, OPFV, and CLC had significantly negative correlations with MSF under warming. However, correlations between the other CPS and coverage changes under warming and cooling were nonsignificant (**Table S4** and **Figure 4**).

**Figure 5:**
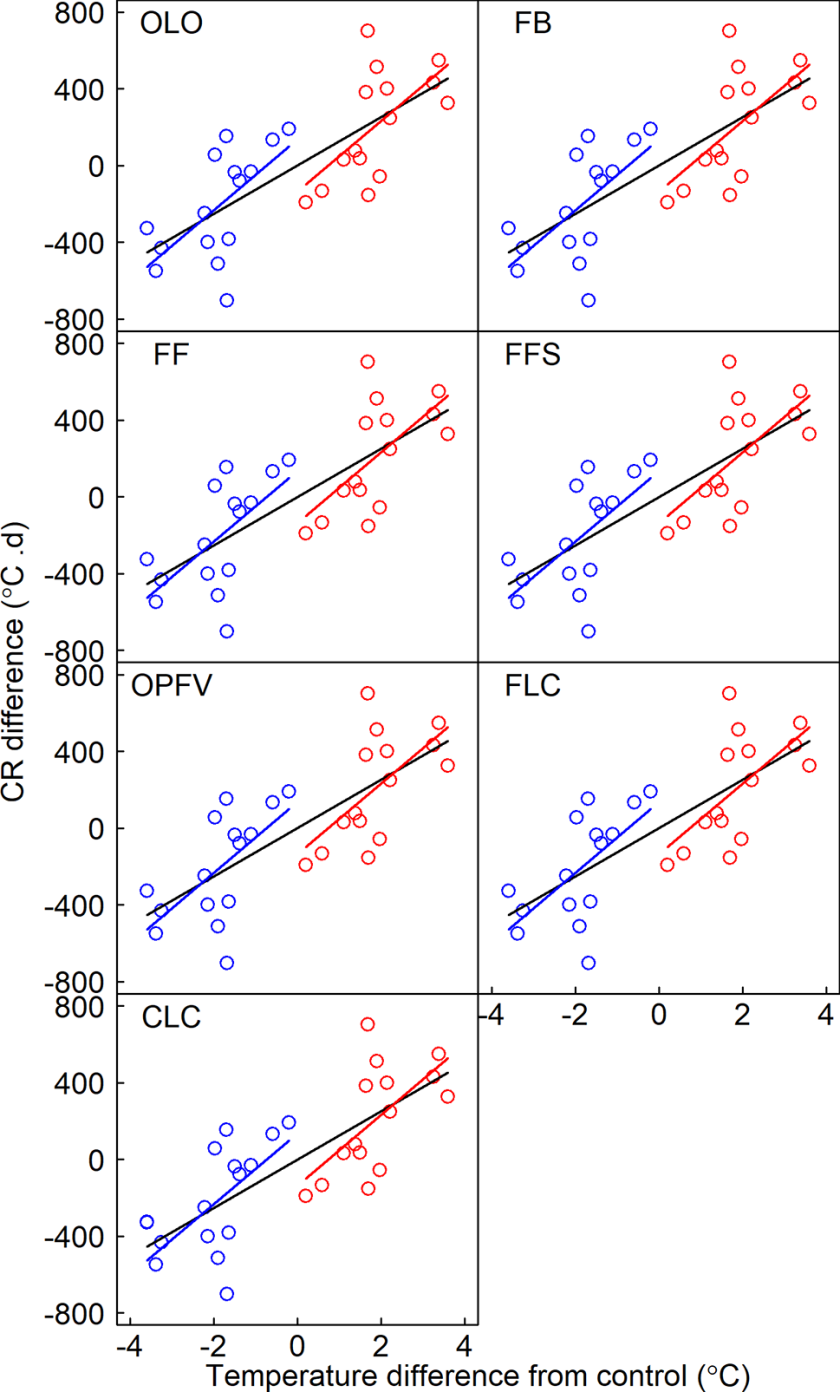
Relationships between differences in chilling requirements (CRs) and the temperature difference between receptor site and donor site. CR is defined as the sum of daily soil mean temperature below 0°C from 1 November of the previous year to a certain phenophase. Linear regression models for warming, cooling, and pooled warming and cooling data are indicated by red, blue, and black lines, respectively. OLO, onset of leaf-out; FB, first bud/boot set; FF, first flowering; FFS, first fruit set for forbs or seeding set for graminoids; OPFV, onset of postfruiting vegetation; FLC, first leaf coloring; CLC, the date of complete leaf coloring.

**Figure 6:**
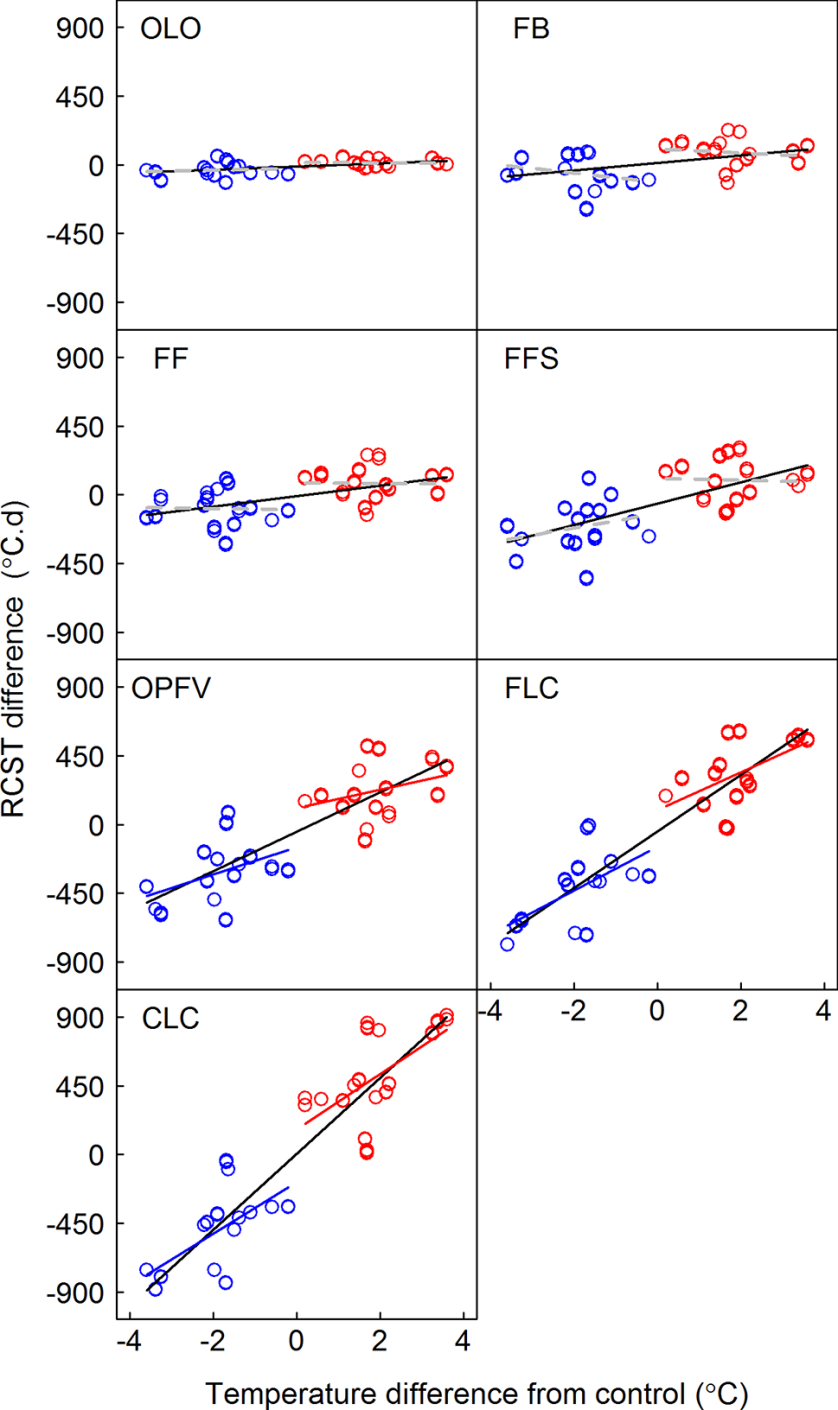
Relationships between differences in required accumulated soil temperature (RCST) and the temperature difference between receptor site and donor site. Linear regression models for warming, cooling, and pooled warming and cooling data are indicated by red, blue, and black lines, respectively. OLO, onset of leaf-out; FB, first bud/boot set; FF, first flowering; FFS, first fruit set for forbs or seeding set for graminoids; OPFV, onset of postfruiting vegetation; FLC, first leaf coloring; CLC, the date of complete leaf coloring.

Seven phenological sequences have the same chilling accumulations (i.e., CR) after transfer (**Figure 5**). The temperature sensitivities of RCST of three early community phenophases (i.e., OLO, FB, and FF) and linear regressions of FFS were nonsignificant under warming and cooling. However, the temperature sensitivities of CR for all seven CPS and RCST in late CPS (i.e., OPFV, FLC, and CLC) had symmetric responses to warming and cooling (**Tables S4 and S5**, **Figures 5** and **6**).

## Discussion

Our reciprocal transplant experiment allows us to distinguish the effects of induced warming and cooling on plant phenophases. Interestingly, we found that the temperature sensitivities of change in ESF and MSF coverage and the temperature sensitivities in flowering stages (FB and FF) had asymmetric responses to warming and cooling, whereas the temperature sensitivities of vegetative phenophases and FFS had symmetric responses to warming and cooling. We propose two explanations involving biotic and abiotic factors that determine symmetric or asymmetric responses of the temperature sensitivities of CPS to warming and cooling.

### Effects of Biotic Factors

Three biotic mechanisms may explain the symmetric or asymmetric responses of CPS to warming and cooling in our study. First, many studies have shown that warming significantly changes species and functional group composition (Walker et al., 2006; Chuine, 2010; Wang et al., 2012). In our study, we found that responses of coverage of ESF and MSF plant groups had contrasting trends. This may be caused by interspecific competition. For example, ESF were relatively short and MSF were relatively tall, and warming promoted tall plants. However, increased frequencies of tall plants would aggravate shelter effects on short plants. Cooling, however, would have the opposite effects. Coverage changes of different FFGs may alter the response magnitude of CPS to warming and cooling. Our previous results showed that simple correlation between FFG coverage change and differences in CPS was significant (**Table 3** in Meng et al., 2017). We further analyzed their partial correlations to eliminate the effect of temperature (**Figure 4**). Although temperature sensitivities of ESF and MSF were greater to warming than to cooling because of asymmetric responses (**Table 1**; **Figure 2**), in general, the correlation coefficients between FFG coverage changes and differences in CPS were greater under cooling than warming (**Figure 4**). Thus, ESF or MSF coverage change gave rise to symmetric responses of CPS to warming and cooling. Therefore, OLO, OPFV, and CLC had symmetric responses to warming and cooling due to their significant relationships with FFG coverage change under warming and cooling (**Table 2**; **Figures 3** and **4**). FFS and FLC also had symmetric responses due to the effects of increased coverage of ESF and decreased coverage of MSF under cooling (**Table 2**; **Figures 3** and **4**).

However, our results showed that temperature sensitivities in the flowering stages had asymmetric responses to warming and cooling. Although coverage change of ESF under warming and cooling caused symmetric responses of CPS, decreased coverage of MSF under cooling significantly delayed CPS due to their significant relationships (**Figure 4**). Therefore, coverage changes of ESF and MSF caused asymmetric responses to warming and cooling in flowering stages, and flowering stages were more sensitive to cooling (**Table 2**; **Figure 3**). Such a response could prevent vulnerable reproductive tissue from being damaged due to low temperatures in spring (Körner et al., 2016; Körner and Basler, 2010; Inouye, 2008) because reproductive phenophases are the most important phases determining population dynamics (Hoffmann et al., 2010; Craine et al., 2012). These results show that changes in species richness and abundance in a community could affect the responses of CPS to warming and cooling. Therefore, ignoring changes in community composition when studying CPS would lead to inaccurate predictions.

Second, different flowering species and functional groups may have compensatory effects on CPS. In general, the vegetative stage and FFS of different species and FFGs had divergent responses to warming and cooling, with both asymmetric and symmetric responses (**Table S6**, Wang et al., 2014a). A time niche complementarity effect between species and FFGs may be associated with symmetric responses to warming and cooling (**Table S6**). This may indicate that different hierarchical levels in an ecosystem have different response characteristics and that symmetric responses at higher (e.g., community) levels are more stable compared with responses at lower (e.g., species or population) levels, which is consistent with previous phenological hierarchy theory (Li et al., 2016). We found that the temperature sensitivities in the flowering stage (FB and FF) of different flowering species and functional groups had asymmetric responses to warming and cooling (**Table S6**). Moreover, the temperature sensitivities of flowering stages for ESF and MSF were greater under cooling than under warming (6.7 vs. -5.1°C, Wang et al., 2014a). Therefore, divergent responses of species and different FFGs caused asymmetric responses to warming and cooling at the community level.

Third, pollinator availability is considered to be the most important determining factor for flowering phenology (De Jong and Klinkhamer, 1991; Mahoro, 2002; Byers, 2017). Nearly 90% of flowering plants are entomophilous (Shivanna and Tandon, 2014). Therefore, flowering could not continuously advance partly due to loss of pollinators under warming. For example, flowering advance per day would lose 0.31 pollinators under warming (Petanidou et al., 2014). However, under cooling, plants only need to postpone flowering to wait for the suitable temperature and for pollinators to come (Kudo and Ida, 2013). Thus, asymmetric responses may be attributed to avoiding a mismatch between pollinators and plant (Rafferty and Ives, 2011; Kudo and Ida, 2013). However, there were no such constraints due to symbiotic relationships for vegetative phenophases and FFS. Therefore, these phenophases showed symmetric responses to warming and cooling.

### Effects of Abiotic Factors

Many studies show that chilling and heat requirements trigger the onset of plant phenology (e.g., Fu et al., 2015; Cong et al., 2017b), and they are key variables in phenological models (Chuine et al., 2000; Schwartz, 2003; Richardson et al., 2013). Our results show that chilling accumulations remained unchanged (**Figure 5**) because daily average temperature is above 0°C after OLO. Meanwhile, chilling accumulations of phenophases have symmetric responses to warming and cooling (**Figure 5**). Therefore, CRs may be not the factor leading to divergent effects on flowering and vegetative processes. However, our results showed that the temperature sensitivities of RCST in early CPS (i.e., OLO, FB, FF, and FFS) had a nonsignificant linear regression model with warming and cooling (**Figure 6**). The response mode of RCST did not match with early CPS (**Figures 3** and **6**). This may be caused by RCST in early CPS being affected by many factors, such as chilling accumulation (Fu et al., 2015). Such effects could decrease the risk of spring cold damage in order to increase plant fitness (Bagnall and Wolfe, 1978; Inouye, 2008; Post et al., 2008; Hacker et al., 2011; Haggerty and Galloway, 2011). However, we found that the temperature sensitivities of RCST in late CPS (i.e., OPFV, FLC, and CLC) had symmetric responses to warming and cooling (**Figure 6**). Their response mode matched with that of early CPS (**Figures 3** and **6**). This could explain symmetric responses of the temperature sensitivities of late CPS due to their close relationships (Franks et al., 2014). This may indicate that late CPS are mainly affected by RCST, whereas early phenophases are less strongly affected by RCST.

### Implications of Symmetric and Asymmetric Responses of CPS to Temperature Change

Our results showed symmetric responses of the temperature sensitivities of community vegetative phenophases to warming and cooling spells (**Table 2**; **Figure 3**). These results suggest that cooling spells have no significant influence on the prediction of vegetative phenophases under a long-term warming trend. Although continued warming could increase heating requirements (**Figure 6**) as in other studies, we found that cooling could symmetrically decrease heating requirements for vegetative phenophases. Therefore, the responses of vegetative phenophases to long-term warming would not be diminished by short-term cooling spells. The decreased magnitude of the response of vegetative phenophases may be attributed to increased heating requirements (Fu et al., 2014; Fu et al., 2015) or increased heat requirement beyond the temperature increase (Cong et al., 2017a). However, it is noteworthy that different ecosystems may show different symmetric or asymmetric responses to warming and cooling. For example, Wolkovich et al. (2012) found that the temperature sensitivity of all pooled species observed under warming experiments is underestimated compared with long-term observations. This difference may be caused by asymmetric responses to warming and cooling due to pooling all species from different regions (**Figure 2** in Wolkovich et al., 2012). In particular, species richness and abundance would be altered under rapid warming (Richardson et al., 2013; Polgar et al., 2014; Tang et al., 2016). This would change the response pattern to warming and cooling in the future. We found that, unlike vegetative phenophases, reproductive phenophases had asymmetric responses to warming and cooling, and that they were more sensitive to cooling (**Table 2** and **Figure 3**). Therefore, pooled warming and cooling data would underestimate the responses of reproductive phenophases to warming. Hence, studies on reproductive phenophases should distinguish the effects of warming and cooling because asymmetric responses would mask the effects of warming or cooling.

## Conclusions

Our results showed that coverage changes of FFGs and RCST codetermined the asymmetric (flowering stages) and symmetric responses (vegetative phenophases) of CPS to warming and cooling. Therefore, if data from warming and cooling periods under a long-term warming trend are pooled, reproductive phenophases would be underrated due to a higher effect size under cooling. Although our reciprocal transfer experiments could distinguish the different effect sizes of warming and cooling, only alpine herbaceous species were monitored, and we just conducted the experiment for 3 years. We therefore suggest that more reciprocal transfer experiments are conducted or new technologies developed to distinguish warming and cooling effects on herbaceous and woody species.

## Data Availability Statement

All datasets generated for this study are included in the manuscript/**Supplementary Files**.

## Author Contributions

SW designed this experiment; FM, JS, LZ, ZZ, QW, BL, WL, LJ, JD, PL, WR, CL, and MD performed this experiment; FM, HN, SW, ZW, and TD analyzed all data; FM, SW, and HN wrote this manuscript, and all persons provided some comments and suggestions for the manuscript.

## Funding

This work was supported by projects from the National Science Foundation of China (41230750, 31672470, and 31470524), the “National Key Research and Development Program of China” (2016YFC0501802), the China Postdoctoral Science Foundation (2017LH033 and 2018M640187), and the National Natural Science Foundation for the Youth of China (31702162).

## Conflict of Interest

The authors declare that the research was conducted in the absence of any commercial or financial relationships that could be construed as a potential conflict of interest.
