# Supplementary material for: Divergent Responses of Community Reproductive and Vegetative Phenology to Warming and Cooling: Asymmetry Versus Symmetry
Source: Front Plant Sci. 2019 Oct 17;10:1310. doi: 10.3389/fpls.2019.01310 (PMC6811613; doi:10.3389/fpls.2019.01310)
Supplement: Supplementary file 1 [file Table_1.docx]

**SUPPLEMENTARY MATERIALS:**

**TABLE S1 |** All species from the same original elevation for all transferred plots.

| Flower type | 3200 m | 3400 m | 3600 m | 3800 m |
| --- | --- | --- | --- | --- |
| ESF | *Anemone cathayensis* | *Anemone cathayensis* | *Anemone cathayensis* | *Anemone cathayensis* |
| ESF | *Aster yunnanensis* | *Aster yunnanensis* | *Aster yunnanensis* | *Aster yunnanensis* |
| ESF |  |  |  | *Cardamine tangutorum* |
| ESF | *Carex scabrirostris* | *Carex scabrirostris* | *Carex scabrirostris* | *Carex scabrirostris* |
| ESF |  |  | *Gentiana farreri* |  |
| ESF |  | *Glaux maritima* |  | *Glaux maritima* |
| ESF | *Halerpestes tricuspis* | *Halerpestes tricuspis* | *Halerpestes tricuspis* | *Halerpestes tricuspis* |
| ESF | *Kobresia humilis* | *Kobresia humilis* | *Kobresia humilis* | *Kobresia humilis* |
| ESF | *Lancea tibetica* | *Lancea tibetica* | *Lancea tibetica* | *Lancea tibetica* |
| ESF |  | *Microula sikkimensis* |  |  |
| ESF | *Polygonum viviparum* | *Polygonum viviparum* | *Polygonum viviparum* | *Polygonum viviparum* |
| ESF | *Taraxacum mongolicum* | *Taraxacum mongolicum* | *Taraxacum mongolicum* | *Taraxacum mongolicum* |
| ESF |  |  | *Thalictrum alpinum* | *Thalictrum alpinum* |
| ESF | *Festuca ovina* | *Festuca ovina* | *Festuca ovina* | *Festuca ovina* |
| ESF | *Iris potaninii* | *Iris potaninii* |  |  |
| ESF | *Poa annua* | *Poa annua* | *Poa annua* | *Poa annua* |
| ESF | *Potentilla bifurca* | *Potentilla bifurca* | *Potentilla bifurca* | *Potentilla bifurca* |
| ESF | *Gueldenstaedtia diversifolia* | *Gueldenstaedtia diversifolia* | *Gueldenstaedtia diversifolia* | *Gueldenstaedtia diversifolia* |
| ESF |  |  | *Kobresia capillifolia* | *Kobresia capillifolia* |
| ESF | *Stellaria umbellata* | *Stellaria umbellata* | *Stellaria umbellata* | *Stellaria umbellata* |
| ESF | *Corydalis pallida* |  | *Corydalis pallida* | *Corydalis pallida* |
| ESF | *Gentiana scabra* | *Gentiana scabra* | *Gentiana scabra* | *Gentiana scabra* |
| ESF | *Helictotrichon tibeticum* | *Helictotrichon tibeticum* | *Helictotrichon tibeticum* |  |
| ESF | *Koeleria cristata* | *Koeleria cristata* | *Koeleria cristata* | *Koeleria cristata* |
| ESF | *Astragalus membranaceus* |  |  |  |
| ESF | *Veronica ciliata* | *Veronica ciliata* | *Veronica ciliata* |  |
| ESF |  |  | *Notopterygium incisum* |  |
| ESF |  |  |  | *Poa bomiensis* |
| ESF |  | *Chrysosplenium pilosum* | *Chrysosplenium pilosum* | *Chrysosplenium pilosum* |
| ESF | *Festuca rubra* | *Festuca rubra* | *Festuca rubra* |  |
| ESF | *Plantago depressa* |  |  |  |
| ESF | *Pomatosace filicula* |  |  |  |
| MSF |  |  |  | *Carex atrofusca* |
| MSF | *Elymus nutans* | *Elymus nutans* | *Elymus nutans* | *Elymus nutans* |
| MSF | *Euphrasia pectinata* | *Euphrasia pectinata* |  |  |
| MSF | *Gentiana grumii* | *Gentiana grumii* | *Gentiana grumii* |  |
| MSF | *Gentiana straminea* | *Gentiana straminea* |  |  |
| MSF | *Geranium pylzowianum* | *Geranium pylzowianum* | *Geranium pylzowianum* | *Geranium pylzowianum* |
| MSF |  | *Halenia corniculata* | *Halenia corniculata* | *Halenia corniculata* |
| MSF |  |  | *Leontopodium pusillum* | *Leontopodium pusillum* |
| MSF | *Ligularia virgaurea* | *Ligularia virgaurea* | *Ligularia virgaurea* | *Ligularia virgaurea* |
| MSF | *Medicago ruthenica* | *Medicago ruthenica* | *Medicago ruthenica* | *Medicago ruthenica* |
| MSF | *Oxytropis deflexa* | *Oxytropis deflexa* |  |  |
| MSF |  | *Oxytropis kansuensis* |  |  |
| MSF |  | *Oxytropis ochrocephala* |  |  |
| MSF | *Pedicularis kansuensis* | *Pedicularis kansuensis* | *Pedicularis kansuensis* | *Pedicularis kansuensis* |
| MSF |  | *Poa alpigena* | *Poa alpigena* | *Poa alpigena* |
| MSF | *Poa pratensis* | *Poa pratensis* | *Poa pratensis* | *Poa pratensis* |
| MSF | *Polygonum sibiricum* | *Polygonum sibiricum* | *Polygonum sibiricum* | *Polygonum sibiricum* |
| MSF | *Potentilla anserina* | *Potentilla anserina* | *Potentilla anserina* | *Potentilla anserina* |
| MSF | *Potentilla fruticosa* | *Potentilla fruticosa* | *Potentilla fruticosa* | *Potentilla fruticosa* |
| MSF | *Potentilla nivea* | *Potentilla nivea* | *Potentilla nivea* | *Potentilla nivea* |
| MSF |  | *Rheum pumilum* | *Rheum pumilum* | *Rheum pumilum* |
| MSF |  |  | *Rumex crispus* |  |
| MSF |  |  | *Saussurea katochaete* | *Saussurea katochaete* |
| MSF |  |  |  | *Saxifraga atrata* |
| MSF | *Scirpus distigmaticus* | *Scirpus distigmaticus* | *Scirpus distigmaticus* | *Scirpus distigmaticus* |
| MSF | *Stipa aliena* | *Stipa aliena* | *Stipa aliena* | *Stipa aliena* |
| MSF | *Thalictrum alpinum* | *Thalictrum alpinum* | *Thalictrum alpinum* | *Thalictrum alpinum* |
| MSF |  | *Trollius farreri* |  |  |
| MSF | *Gentianella Moench* | *Gentianella Moench* | *Gentianella Moench* | *Gentianella Moench* |
| MSF | *Leontopodium nanum* | *Leontopodium nanum* | *Leontopodium nanum* | *Leontopodium nanum* |
| MSF |  |  | *Euphrasia regelii* | *Euphrasia regelii* |
| MSF | *Oxytropis* | *Oxytropis* | *Oxytropis* | *Oxytropis* |
| MSF | *Elsholtzia calycocarpa* | *Elsholtzia calycocarpa* | *Elsholtzia calycocarpa* |  |
| MSF | *Comastoma pulmonarium* | *Comastoma pulmonarium* | *Comastoma pulmonarium* | *Comastoma pulmonarium* |
| MSF |  | *Notopterygium incisum* | *Notopterygium incisum* |  |
| MSF | *Morina chinensis* |  | *Morina chinensis* |  |
| MSF | *Deschampsia caespitosa* | *Deschampsia caespitosa* |  |  |
| MSF | *Humulus japonicus* | *Humulus japonicus* |  |  |
| MSF |  |  | *Entire Meconopsis* |  |
| LAF |  | *Allium cyaneum* |  |  |
| LAF | *Gentiana farreri* | *Gentiana farreri* | *Gentiana farreri* | *Gentiana farreri* |
| LAF | *Saussurea pulchra* | *Saussurea pulchra* | *Saussurea pulchra* | *Saussurea pulchra* |
| LAF | *Saussurea nigrescens* | *Saussurea nigrescens* | *Saussurea nigrescens* | *Saussurea nigrescens* |
| LAF |  |  |  | *Corydalis pallida* |
| LAF | *Lomatogonium rotatum* | *Lomatogonium rotatum* | *Lomatogonium rotatum* |  |

Note: Three functional flowering groups including early-spring (ESF), mid-summer (MSF) and late-autumn flowering species (LAF), respectively.

**TABLE S2 |** Pearson’s correlations between community phenological sequences and annual air/soil temperatures across all treatments.

| Temperature | OLO | FB | FF | FFS | OPFV | FLC | CLC |
| --- | --- | --- | --- | --- | --- | --- | --- |
| Air-Tem | -0.471** | -0.462** | -0.348** | -0.312** | -0.259** | -0.005 | 0.521** |
| Soil-Tem | -0.681** | -0.646** | -0.599** | -0.643** | -0.496** | -0.355** | 0.491** |

**TABLE S3 |** Coefficients of regression models (y=*a*x+*b*) of Fig 1. “**—**” signifies no values.

|  | Treats | *a* | *b* | *P* | R^2^ |
| --- | --- | --- | --- | --- | --- |
| ESF | Pooled | -3.350 | -1.267 | <0.001 | 0.278 |
|  | Warming | -10.422 | 14.752 | <0.001 | 0.347 |
|  | Cooling | -4.345 | -5.097 | 0.001 | 0.241 |
| MSF | Pooled | 3.390 | 1.434 | <0.001 | 0.275 |
|  | Warming | 10.656 | -14.914 | <0.001 | 0.352 |
|  | Cooling | 3.930 | 4.292 | 0.005 | 0.186 |
| LAF | Pooled | — | — | — | — |
|  | Warming | — | — | — | — |
|  | Cooling | — | — | — | — |
| OLO | Pooled | -8.537 | -0.544 | <0.001 | 0.918 |
|  | Warming | -5.752 | -7.034 | <0.001 | 0.630 |
|  | Cooling | -5.870 | 5.722 | <0.001 | 0.535 |
| FB | Pooled | -8.546 | 2.044 | <0.001 | 0.918 |
|  | Warming | -7.578 | 0.406 | <0.001 | 0.778 |
|  | Cooling | -10.325 | -1.492 | <0.001 | 0.691 |
| FF | Pooled | -8.474 | 0.556 | <0.001 | 0.929 |
|  | Warming | -6.739 | -2.974 | <0.001 | 0.680 |
|  | Cooling | -9.063 | -0.292 | <0.001 | 0.717 |
| FFS | Pooled | -8.605 | -2.967 | <0.001 | 0.801 |
|  | Warming | -8.270 | -3.868 | <0.001 | 0.614 |
|  | Cooling | -7.750 | -1.086 | <0.001 | 0.278 |
| OPFV | Pooled | -5.633 | -2.344 | <0.001 | 0.730 |
|  | Warming | -4.081 | -5.602 | <0.001 | 0.235 |
|  | Cooling | -5.713 | -2.161 | <0.001 | 0.314 |
| FLC | Pooled | -3.667 | -2.656 | <0.001 | 0.663 |
|  | Warming | -1.284 | -7.715 | <0.001 | 0.044 |
|  | Cooling | -3.546 | -1.856 | 0.001 | 0.226 |
| CLC | Pooled | 4.294 | -2.122 | <0.001 | 0.780 |
|  | Warming | 3.057 | 0.775 | <0.001 | 0.261 |
|  | Cooling | 3.044 | -5.044 | <0.001 | 0.259 |

Note: ESF: early-spring flowering functional group; MSF: mid-summer flowering functional group; LAF: late-autumn flowering functional group. OLO: onset of leaf-out; FB: first bud/boot-set; FF: first flowering; FFS: first fruit-set for forbs or seeding-set for graminoids; OPFV: onset of post-fruiting vegetation; FLC: first leaf coloring and CLC: the date of complete leaf-coloring.

**TABLE S4 |** Slope heterogeneity of temperature sensitivities of chilling requirements (CR) and required accumulated soil temperature (RCST) in different community phenological sequences between warming and cooling. “**—**” signifies no values.

|  | OLO | FB | FF | FFS | OPFV | FLC | CLC |
| --- | --- | --- | --- | --- | --- | --- | --- |
| CR | 0.999 | 0.999 | 0.999 | 0.999 | 0.999 | 0.999 | 0.999 |
| RCST | **—** | **—** | **—** | **—** | 0.478 | 0.642 | 0.771 |

Note: OLO: onset of leaf-out; FB: first bud/boot-set; FF: first flowering; FFS: first fruit-set for forbs or seeding-set for graminoids; OPFV: onset of post-fruiting vegetation; FLC: first leaf coloring and CLC: the date of complete leaf-coloring.

**TABLE S5 |** Coefficients of regression models (y=*a*x+*b*) of Fig 5. “**—**” signifies no values.

| Phenophases | Treats | *a* | *b* | *P* | R^2^ |
| --- | --- | --- | --- | --- | --- |
| OLO | Pooled | 10.560 | -11.431 | <0.001 | 0.250 |
|  | Warming | **—** | **—** | **—** | **—** |
|  | Cooling | **—** | **—** | **—** | **—** |
| FB | Pooled | 24.662 | 11.300 | <0.001 | 0.180 |
|  | Warming | **—** | **—** | **—** | **—** |
|  | Cooling | **—** | **—** | **—** | **—** |
| FF | Pooled | 34.786 | -8.876 | <0.001 | 0.280 |
|  | Warming | **—** | **—** | **—** | **—** |
|  | Cooling | **—** | **—** | **—** | **—** |
| FFS | Pooled | 69.872 | -58.605 | <0.001 | 0.470 |
|  | Warming | **—** | **—** | **—** | **—** |
|  | Cooling | **—** | **—** | **—** | **—** |
| OPFV | Pooled | 129.171 | -47.704 | <0.001 | 0.690 |
|  | Warming | 60.023 | 107.540 | 0.036 | 0.080 |
|  | Cooling | 88.640 | -149.049 | 0.004 | 0.160 |
| FLC | Pooled | 185.124 | -43.144 | <0.001 | 0.800 |
|  | Warming | 122.921 | 97.806 | <0.001 | 0.276 |
|  | Cooling | 142.970 | -146.333 | <0.001 | 0.307 |
| CLC | Pooled | 248.467 | 4.041 | <0.001 | 0.850 |
|  | Warming | 181.837 | 163.066 | <0.001 | 0.380 |
|  | Cooling | 168.033 | -180.984 | <0.001 | 0.371 |

Note: OLO: onset of leaf-out; FB: first bud/boot-set; FF: first flowering; FFS: first fruit-set for forbs or seeding-set for graminoids; OPFV: onset of post-fruiting vegetation; FLC: first leaf coloring and CLC: the date of complete leaf-coloring.

**TABLE S6 |** Slope heterogeneity of the temperature sensitivities of six common species between warming and cooling. “**—**” signifies *P* values of species first flowering date are in Wang et al. (2014a).

|  | OLO | FB | FF | FFS | OPFV | FLC | CLC |
| --- | --- | --- | --- | --- | --- | --- | --- |
| ESF | 0.274 | **<0.001** | **<0.001** | **<0.001** | **<0.001** | 0.487 | **0.021** |
| *Kobresia humilis* | 0.492 | **0.002** | **—** | **<0.001** | **<0.001** | 0.932 | 0.309 |
| *Carex scabrirostris* | 0.395 | **<0.001** | **—** | **<0.001** | **<0.001** | 0.427 | **0.013** |
| MSF | **<0.001** | **0.012** | **<0.001** | 0.051 | **0.001** | **0.001** | **<0.001** |
| *Potentilla anserine* | **0.001** | 0.215 | **—** | 0.472 | **0.007** | **<0.001** | **0.001** |
| *P. nivea* | **0.008** | **0.001** | **—** | 0.568 | **0.028** | **<0.001** | **0.006** |
| *Poa pratensis* | 0.165 | 0.532 | **—** | 0.129 | 0.271 | 0.889 | 0.722 |
| *Stipa aliena* | 0.131 | 0.331 | **—** | **0.027** | 0.408 | 0.944 | **0.450** |

Notes: *P* value in the ANCOVA method is the interaction effect in the test of the linear model. OLO: onset of leaf-out; FB: first bud/boot-set; FF: first flowering; FFS: first fruit-set for forbs or seeding-set for graminoids; OPFV: onset of post-fruiting vegetation; FLC: first leaf coloring and CLC: the date of complete leaf-coloring. ESF here uses pooled data on *Kobresia humilis* and *Carex scabrirostris*, and MSF uses pooled data on *Potentilla anserine*, *P. nivea, Poa pratensis* and *Stipa aliena*.
